# Case presentation of delayed superficial femoral artery pseudoaneurysm causing an acute deep vein thrombosis

**DOI:** 10.1016/j.jvscit.2022.05.012

**Published:** 2022-07-02

**Authors:** Max Murray-Ramcharan, Syed Ali Raza Rizvi

**Affiliations:** a Division of Vascular Surgery, Department of Surgery, Harlem Hospital Center, Columbia University, New York, NY; b Department of Surgery, Harlem Hospital Center, Columbia University, New York, NY

**Keywords:** Deep vein thrombosis, Delayed presentation, Lower extremity, Mass effect, Open surgery, Pseudoaneurysm, Vascular closure device

## Abstract

In the present report, we have described the case of a significantly delayed presentation of a pseudoaneurysm (PSA) and subsequent mass effect causing an acute deep vein thrombosis (DVT). The patient had presented with a mass in the right groin and edema of the right lower extremity prompting further imaging studies. The imaging studies demonstrated a superficial femoral artery PSA and an acute femoral vein DVT. Our patient had no history of recent trauma or femoral access procedures performed in the last ≥5 years. Surgical repair of the PSA was performed, and the DVT was managed with anticoagulation therapy.

A pseudoaneurysm (PSA) refers to the leakage of blood from an artery into the surrounding tissue resulting in a persistent connection to an adjacent cavity. This can occur after a needlestick, vascular intervention, or trauma to an artery, with an incidence of 2.9% to 3.8% in femoral access procedures.^1^ A PSA can present as a swelling or pulsatile mass at the region of arterial manipulation. The most concerning adverse outcome of a PSA has been rupture.

Other complications have included embolism, pain, infection, and local compression of the surrounding structures. If a PSA is suspected clinically, several imaging studies can aid in the diagnostic workup. Ultrasound can provide information such as the location of the PSA in relation to blood vessels, PSA size, compartments within the sac, and the neck diameter. The addition of Doppler ultrasound can show blood flow within the sac and communication with a specific vessel. Computed tomography angiography and magnetic resonance angiography are also useful studies that can characterize further details of the PSA, including its chronicity, possible evidence of infection, and three-dimensional reconstruction of the vascular beds. Treatment of a PSA has included compression alone, direct thrombin injection with ultrasound guidance, endovascular intervention with stenting or embolization, and surgical intervention with repair, bypass, or ligation.^2^

## Case report

A 47-year-old man with a history of end-stage renal disease requiring hemodialysis since 2007 had presented with a mass in the right groin and edema of the right lower extremity. He had a history of anemia requiring multiple admissions without a clear etiology identified. His surgical history included a kidney transplant performed in 2012, which was unsuccessful as of 2014. The patient also had a right arm arteriovenous fistula created for dialysis until his renal transplant, which was used again after transplant rejection. The patient provided written informed consent for the report of his case details and imaging studies.

**Fig 1:**
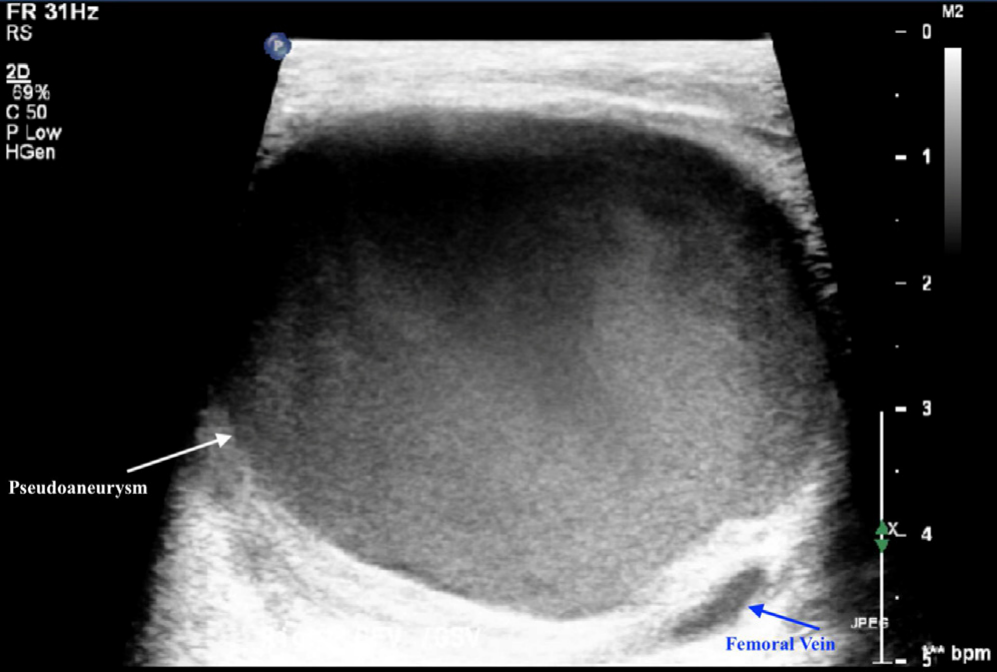
Venous duplex ultrasound showing pseudoaneurysm and femoral vein.

**Fig 2:**
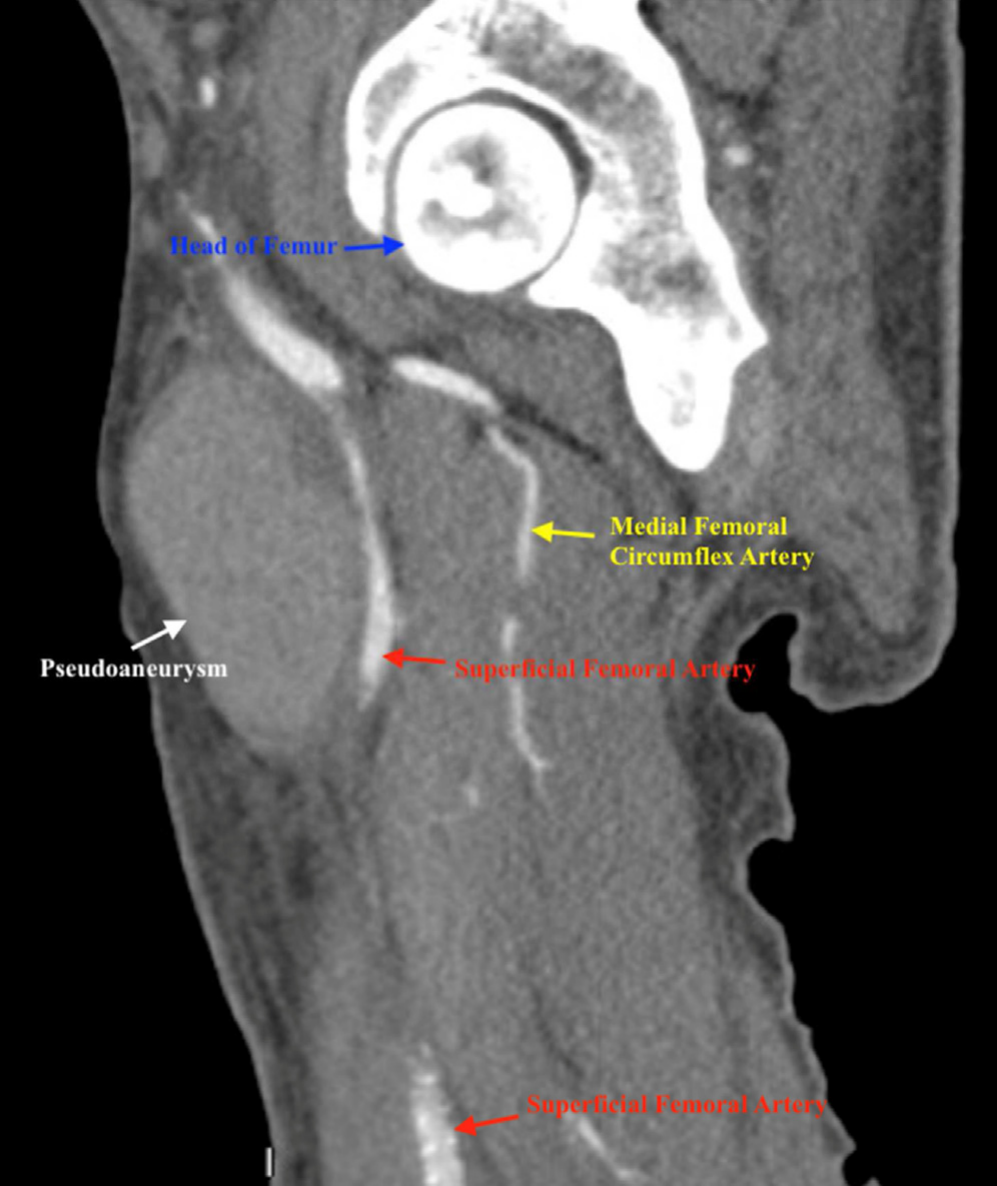
Computed tomography angiogram showing pseudoaneurysm, superficial femoral artery, and medial femoral circumflex artery.

**Fig 3:**
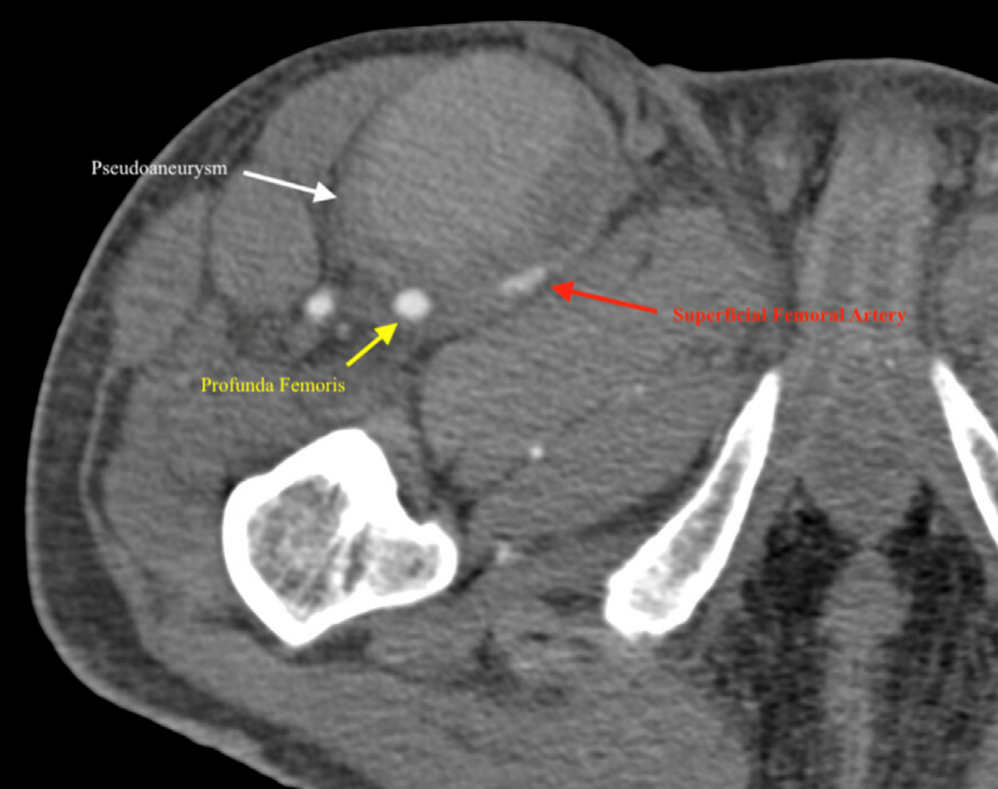
Computed tomography angiogram showing pseudoaneurysm, superficial femoral artery, and profunda femoris.

The patient had been admitted for medical management of anemia (hemoglobin, 6.5 g/dL) found on routine laboratory tests at his outpatient nephrology appointment. During his hospital stay, his primary team noted that his right thigh and inguinal region was more edematous than was his left. The patient endorsed that the edema was chronic and had been present for months. Venous duplex ultrasound of the right thigh revealed a PSA adjacent to the right common femoral artery (CFA) measuring 5.0 × 4.1 × 4.9 cm (Fig 1, Fig 2, Fig 3). The PSA was compressing the femoral vein, resulting in a chronic-appearing deep vein thrombosis (DVT). His primary team began a heparin infusion for anticoagulation therapy, and later the vascular surgery department was consulted for recommendations.

During our interview, the patient denied any pain, numbness, or tingling in the inguinal region. He further denied any trauma, needlesticks, angiographic procedures, or temporary dialysis catheters placed in the femoral vessels in the previous ≥5 years. In addition, he endorsed a remote history of an unknown intervention with vascular access at the right inguinal region ∼10 years previously. However, he was unable to recall any specific details, and a review of his medical records was unrevealing. On physical examination, his right thigh was moderately edematous compared with his left thigh, and a faintly pulsatile mass could be palpated at the medial inguinal region. He had no tenderness throughout his right leg, and no overlying skin changes were present. Pitting edema was noted at the right ankle. However, he had strong distal pulses with no neuromuscular deficits evident.

Computed tomography angiography of the right lower extremity (RLE) was requested, which demonstrated a 7.9- × 6.4- × 4.9-cm partially thrombosed PSA at the level of the bifurcation of the CFA into the superior femoral artery (SFA) and profunda femoris. We requested discontinuation of the anticoagulation therapy. Owing to the large size of the PSA and the associated mass effect of the PSA on the femoral vein, we elected to proceed with operative intervention. The patient underwent surgery the next day.

We began with open exploration of the right inguinal region via a 10-cm vertical incision, with dissection of the SFA, lateral retraction of the sartorius muscle, and preservation of the great saphenous vein. Proximal and distal control of the SFA was obtained using vessel loops and angled DeBakey clamps. The profunda femoris was also identified at its origin and controlled with a vessel loop. After dividing the overlying scar tissue, we encountered the PSA, which measured ∼6 × 5 cm. Among the surrounding scar tissue, we found a retained Prolene suture (Ethicon Inc, Raritan, NJ). We determined that this was likely from a Perclose ProGlide device (Abbott Laboratories, Chicago, IL) used in his described remote vascular intervention. The suture was removed, and we entered the PSA sac, evacuating 150 to 200 mL of blood and clots. We noted a communication between the SFA and the PSA sac with a small amount of back bleeding from the distal SFA. This was repaired primarily with 6-0 Prolene suture (Ethicon Inc). The entirety of the PSA sac was excised and hemostasis obtained. The femoral vein was not assessed intraoperatively in an effort to avoid unnecessary dissection and potential manipulation of the indwelling thrombus. At the conclusion of the procedure, the wound was closed primarily, and a compression dressing was applied. An inferior vena cava filter was not placed preoperatively, because we did not anticipate any acute embolic events from the chronic-appearing DVT.

Our patient’s postoperative course was largely unremarkable. The RLE was examined daily and showed strong distal pulses and no neurovascular deficits. Also, the inguinal swelling showed no evidence of progression, with resolution of the previously identified pulsatile mass.

The patient was given anticoagulation therapy in the form of a heparin infusion for management of the DVT for 3 days. Subsequently, he was transitioned to apixaban, 5 mg every 12 hours, with a 3-month prescription, because this was his first DVT and the offending cause had been treated. At the subsequent follow-up visits, resolution of the swelling associated with his RLE DVT was noted. He maintained his appointments with vascular surgery for 3 months with no significant complications. His DVT was managed by the hematology department. After 3 months, the apixaban was discontinued, with no residual swelling noted in the RLE at the subsequent follow-up appointments.

## Discussion

In the present case report, we have detailed several uncommon presenting features associated with a PSA. First, the findings from the present case have shown that a PSA can develop or be identified with no recent intervention or inciting event. The most feasible etiology for the PSA in our patient was a remote history of femoral artery access >5 years previously. Most cases of PSA developing after arterial puncture will occur within a few days to weeks, and it is very uncommon for this complication to be identified in a significantly delayed fashion as in our patient. During our review of the literature, we identified a case in which a 61-year-old woman had presented with a painful swelling in the left inguinal region, which was later identified as a PSA of the left CFA. She had an arterial puncture performed at the same site 2 years earlier for cardiac catheterization.^3^ An observational study identified 573 patients who had experienced the complication of a PSA after an arterial puncture procedure.^4^ Of these 573 patients, 5 (0.9% incidence) had presented with delayed symptoms.^4^ They had sought to document the incidence of delayed PSA as more common than previously thought. However, they defined the term “delayed” as an interval of 1 to 3 months after the procedure compared with the substantially more indolent progression of 5 to 10 years identified for our patient.

In the present case, a retained Prolene suture (Ethicon Inc) from a closure device was identified intraoperatively and removed. Without substantial evidence to suggest an alternate cause, we inferred that his PSA had formed owing to failure of the vascular closure device (VCD; Perclose ProGlide; Abbott Laboratories) during the patient’s remote procedure. Malkawi et al^5^ reported an incidence of ProGlide failure of ∼2% to 8% with the use of catheter sizes of 6F to 24F. A similar failure rate of 7.6% was reported in a retrospective study by Chen et al.^6^ They had focused on the use of catheters with a size of 16F to 26F.^6^ In a large Cochrane systematic review comparing the use of suture-based VCDs and manual compression for the outcome of PSA formation, the investigators concluded that the difference in the incidence of PSA was not statistically significant (odds ratio [OR], 0.79; 95% confidence interval [CI], 0.25-2.53; *P* = .70).^7^ When the ProGlide device specifically was compared with collagen-based or metal clip-based VCDs, no significant differences in PSA formation was found (OR, 0.30; 95% CI, 0.01-7.39; *P* = .46; and OR, 0.21; 95% CI, 0.01-5.24; *P* = .34, respectively).^7^

In addition, the PSA in the present case was only identified owing to the occurrence of one of the more uncommon complications of this pathology, specifically the delayed development of an adjacent DVT due to local external compression. A very limited number of cases with similar presenting symptoms have been documented in the literature. After an extensive search for DVTs with an adjacent PSA as the etiology, we identified four case reports with a total of only five patients with a presentation similar to that of our patient.^8^, ^9^, ^10^, ^11^ The duration after the arterial puncture procedure to the diagnosis of DVT was 5 to 14 days. However, one of the cases could not be evaluated, because the etiologies of the PSA and DVT were unknown, with no obvious inciting event reported.^10^ VCDs had been used in two of the initial access procedures, with no VCD used in the other three. These reports had described the formation of an acute DVT, in contrast to the present case of a chronic DVT.

## Conclusions

We have presented the case of a patient with a significantly delayed PSA formed >5 to 10 years after an unknown arterial access procedure. Our patient had presented with a mass effect on the adjacent femoral vein that had resulted in the formation of a chronic DVT. Operative exploration revealed that a ProGlide (Abbott Laboratories) VCD, which had been used in a vascular access procedure ≥5 years earlier, with presumed failure, was a likely factor contributing to PSA formation. A careful review of the imaging studies is required in the evaluation of PSA, particularly the evaluation of the adjacent veins to identify evidence of DVT formation or a mass effect. In the present patient, surgical repair of the PSA was performed, and the DVT was managed with anticoagulation therapy with no long-term complications and a good therapeutic result.
